# Dilated cardiomyopathy complicated by an intracavitary thrombus and acute heart failure: A rare presentation of systemic lupus erythematosus

**DOI:** 10.1016/j.amsu.2022.104739

**Published:** 2022-09-22

**Authors:** Lamyae Zinoune, Mosaab Maazouzi, Karima Benbouchta, Saida Amaqdouf, Noha El ouafi, Nabila Ismaili

**Affiliations:** a Faculty of Medicine and Pharmacy, Mohammed I^st^ University, Oujda, Morocco; b Department of Cardiology, Mohammed VI University Hospital/Mohammed I University, Oujda, Morocco; c Laboratory of Epidemiology, Clinical Research and Public Health, Faculty of Medicine and Pharmacy, Oujda, Morocco

**Keywords:** Heart failure, Dilated cardiomyopathy, Systemic lupus erythematosus, Autoimmune disorder, Lupus cardiomyopathy

## Abstract

**Introduction:**

Cardiac manifestations during systemic lupus erythematosus (SLE) are diverse and often have major prognostic consequences. Lupus cardiomyopathy is an uncommon event in the course of SLE and initial clinical manifestation as decompensated dilated cardiomyopathy is very rare.

**Case report:**

we report the case of a 52-years-old female who presented with acute onset decompensated dilated cardiomyopathy as the initial feature of SLE. The diagnosis was based on clinical, electrocardiographic, angiographic and biochemical characteristics.

**Conclusion:**

Although rare, SLE cardiomyopathy deserves the attention due to its infrequent clinical presentation. It is a complex disease that requires prompt investigation and treatment, otherwise the damage is unrecoverable.

## Highlights

• Lupus cardiomyopathy is a rare entity and usually late clinical manifestation of Systemic Lupus Erythematosus that has been rarely reported in the literature.
• Diagnosis is based on clinical manifestations, electrocardiographic, echocardiographic, angiographic and biochemical characteristics.
• Lupus cardiomyopathy is a complex disease with multiple possible pathological processes that requires prompt investigation and treatment, otherwise the damage is unrecoverable.

## 1 Introduction

Systemic lupus erythematosus (SLE) is a chronic autoimmune disorder affecting principally young women, characterized by inflammation and damage to multiple organ system including the heart [1,2].

SLE cardiomyopathy is uncommon, limited to case reports. It is an infrequent but serious clinical manifestation occurring in up to 9% of SLE patients [3]. Most patients are asymptomatic, however, SLE cardiomyopathy can lead to various complications, including heart failure and arrhythmias [4]. The gold standard for the diagnosis is endomyocardial biopsy [5]. However, with advances in noninvasive cardiac imaging, cardiac magnetic resonance has emerged as the alternative modality owing to the high spatial and temporal resolution and short acquisition time [6]. The management of SLE cardiomyopathy represents a real challenge because of its rarity, therefore, it is important to take a multidisciplinary approach.

## 2 Case presentation

A 52-years-old non-smoker female, with a past medical history of undocumented and unexplored heart disease for 15 years and recurrent miscarriages, presented to our department for progressive dyspnea on exertion (New York Heart Association class III) with orthopnea. Her symptoms developed 4 month prior to presentation but worsened 1 week prior to admission.

On presentation, the patient was dyspneic and tachycardic at rest. The oral temperature was 36.5 °C, the blood pressure was 110/60 mmHg. Examination revealed marked jugular venous, distension, a distended and globally tender abdomen with positive fluid shift, pulmonary bilateral basal rales, as well as marked bilateral lower leg edema.

**Fig. 1:**
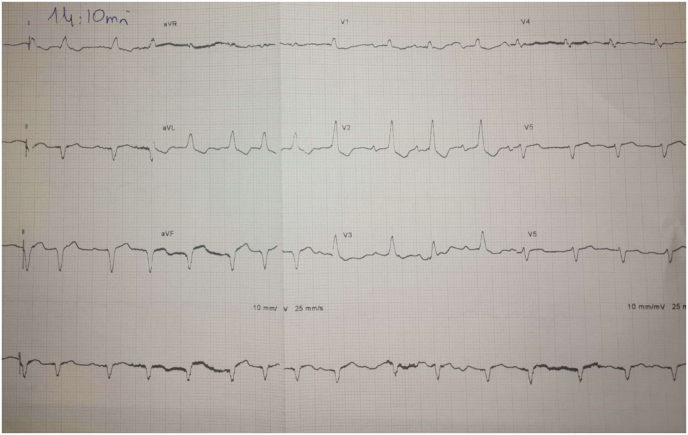
EKG showing left axis deviation with bifascicular block.

An electrocardiogram showed a sinus rhythm with a heart rate of 94 b.p.m, left axis deviation with bifascicular block (right bundle branch block with left anterior fascicular block) (Fig. 1).

**Fig. 2:**
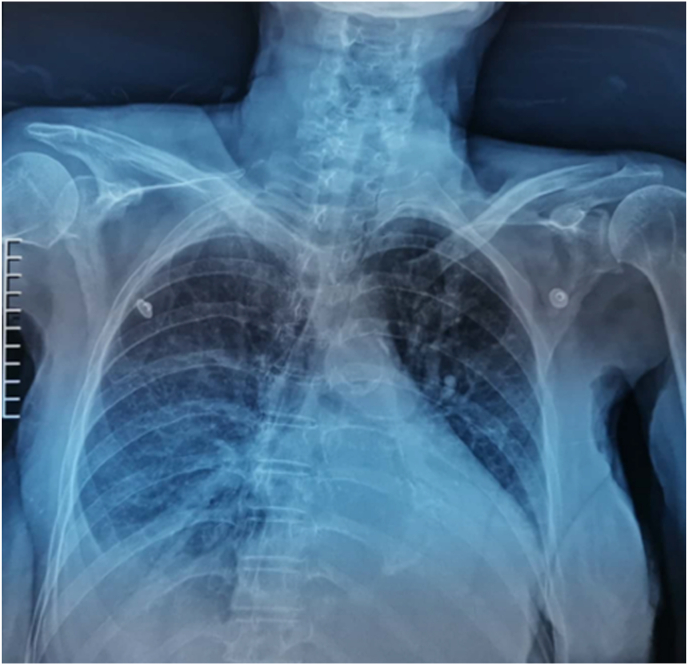
Chest x-ray showing enlargement of the heart, pulmonary congestion and a small right pleural effusion.

Chest x-ray showed symmetrical globular enlargement of the heart and pulmonary congestion in a perihilar distribution, with a small right pleural effusion (Fig. 2).

Her laboratory tests revealed elevated BNP and d-dimers level, lymphopenia and normal Troponin level. Routine blood investigations including coagulation parameters, liver and renal function tests were normal.

**Fig. 3:**
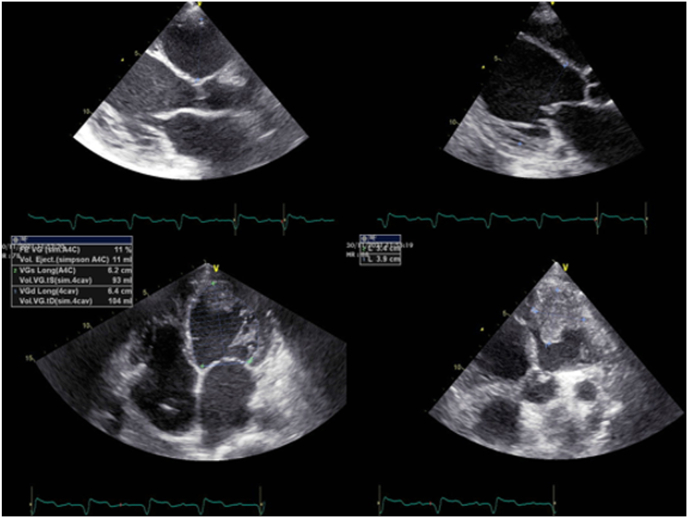
Echocardiogram showing biventricular enlargement with depressed left ventricular ejection fraction and a large apical thrombus.

Echocardiogram revealed a significant biventricular enlargement with diffuse hypokinesis and a markedly depressed left ventricular ejection fraction (LVEF = 11%). A large apical thrombus was noted in the left ventricle. No pericardial effusion and no significant valvulopathy was observed (Fig. 3).

Reversible causes of cardiomyopathy were considered. Chest computed tomography angiography was not supportive of pulmonary embolism as the underlying etiology of right sided heart failure. Laboratory tests revealed normal iron and vitamins levels and normal thyroid function. Serological examination for viral infections, including SARS COV2, herpes simplex, cytomegalovirus, EpsteinBarr, rubeola, hepatitis B and C, and HIV were all normal. A search for underlying malignancy was also negative. Coronary angiography revealed normal coronary arteries with no evidence of stenosis.

The combination of a negative cardiomyopathy screen and the history of recurrent miscarriages, suggested that the most likely etiology was autoimmune. Autoimmune screen showed strongly positive antinuclear antibodies (ANA) at 640 IU/ml (N < 7), with presence of anti-dsDNA antibodies, positive IgM anticardiolipin at 15 IU/ml (N < 10) and IgM anti-β2GP1 antibodies at 14 IU/ml (N < 7). Erythrocyte sedimentation rate, creatininemia, urinary protein excretion, CRP, C3/C4 and CPK were normal.

After a multidisciplinary discussion, the diagnosis of SLE along with antiphospholipid syndrome was made, based on 4 of the 11 criteria of the American College of Rheumatology (pleural effusion, lymphopenia, positive ANA, anti-dsDNA, anticardiolipin and anti-β2GP1 antibodies).

Treatment with prednisone 20mg per day and hydroxychloroquine 400mg per day was begun. The patient was also started on anticoagulation therapy and conventional therapy for heart failure (furosemide, mineralocorticoid receptor antagonist, angiotensin-converting enzyme inhibitors, betablocker and dapagliflozin).

One month later, echocardiography showed only a mild improvement (LVEF = 18%) and unfortunately the patient died few week later at home while sleeping.

## 3 Discussion

Dilated cardiomyopathy (DCM) is a disorder of the cardiac muscle characterized by systolic dilation and dysfunction of one or both ventricles, frequently presenting symptoms of congestive heart failure [7]. A variety of factors may be involved in the pathogenesis of DCM, including viral infections, alcoholism/drugs and toxins, pregnancy and postpartum period, thyrotoxicosis, autoimmune collagen vascular diseases and genetic predisposition [7]. In this patient, most of these possibilities was eliminated, reducing the list of possible etiologies to autoimmune and idiopathic cardiomyopathy. The immunological screen in this case was consistent with a diagnosis of autoimmune vascular disease and, in particular, with systemic lupus erythematosus (SLE).

SLE is an inflammatory autoimmune disease of unknown etiology, affecting most commonly women in childbearing years, that can cause multiple organ damage including the cardiovascular system [1]. The diagnosis of SLE can be very difficult, therefore, the American College of Rheumatology developed 11 diagnostic criteria. The presence of 4 out of 11 criteria allows to establish a definite diagnosis of SLE [8].

Cardiac manifestations during SLE are diverse. Pericarditis is the most frequent form of cardiac involvement, most commonly subclinical noted only on echocardiogram [9]. Myocardial involvement is less common occurring in up to 9% of patients with SLE [10], and is frequently associated with coronary atherosclerosis [11]. Isolated dilated cardiomyopathy, however, is a rare entity and usually late clinical manifestation of SLE that has been rarely reported in the literature [12].

The exact pathogenesis of myocardial involvement is not entirely understood [13]. Case reports of SLE patients with cardiac manifestations who had undergone myocardial biopsy lack evidence for myocarditis [3,14]. This suggest that myocardial inflammation may not be the pathophysiological basis, and that other factors such as thrombotic or inflammatory microvascular coronary disease may play a role [15].

Diagnosis of lupus cardiomyopathy is based on clinical manifestations, electrocardiographic, echocardiographic, angiographic and biochemical characteristics. Coronary angiography is essential to distinguish lupus cardiomyopathy from coronary artery atherosclerosis. However, the gold standard for conforming the diagnosis remains endomyocardial biopsy [5]. This procedure is not routinely used because of its low sensitivity and potential complications [16]. Cardiac magnetic resonance (CMR) is an emerging noninvasive imaging method that may be an alternative to myocardial biopsy [6]. Due to limited access to CMR, our patient did not undergo this test.

There is no consensus on management of SLE cardiomyopathy because of its rarity. Limited evidence supports initial and early management with steroids, conventional therapy for heart failure, adjuvant treatment strategies (anticoagulation and antiarrhythmic drugs) and correction of cardiovascular risk factors [10]. Immunosuppressive and immunoglobulin therapy may be effective in patients in whom active inflammation is detected [17]. In severe cases, cardiac transplant is a viable option for SLE patients with heart failure [18].

## 4 Conclusion

Cardiac involvement in patients with SLE is relatively common and is a significant source of morbidity and mortality. Although rare, SLE cardiomyopathy deserves the attention due to its infrequent clinical presentation. Literature review for SLE presenting as dilated cardiomyopathy revealed a paucity of clinical evidence and consensus. It is a complex disease with multiple possible pathological processes that requires prompt investigation and treatment, otherwise the damage is unrecoverable as proven in our patient.

## Ethical approval

The ethical committee approval was not required given the article type (case report).

The patient gave his informed consent for this case report to be published.

## Funding

This research did not receive any specific grant from funding agencies in the public, commercial, or not-for-profit sectors.

## Author contributions

Lamyae zinoune: study concept, data collection, data analysis, literature research, writing the paper. Mosaab maazouzi: data collection, data analysis. Karima benbouchta: data collection, data analysis. Saida amaqdouf: data collection, data analysis. Noha El ouafi: supervision and data validation. Nabila Ismaili: supervision and data validation.

## Registration of research studies

This is not an original research project involving human participants in an interventional or an observational study but a case report. This registration was not required.

## Consent

The patient gave his informed consent for this case report to be published.

## Guarantor

Lamyae Zinoune.

## Declaration of competing interest

The authors declare no conflicts of interest.
